# The invasome of *Salmonella* Dublin as revealed by whole genome sequencing

**DOI:** 10.1186/s12879-017-2628-x

**Published:** 2017-08-04

**Authors:** Manal Mohammed, Simon Le Hello, Pimlapas Leekitcharoenphon, Rene Hendriksen

**Affiliations:** 1 Department of Biomedical Sciences, Faculty of Science and Technology, University of Westminster, London, UK; 2 Unité des Bactéries Pathogènes Entériques, Centre National de Référence des Salmonella, WHO Collaborating Centre for Reference and Research on Salmonella, Institut Pasteur, Paris, France; 3 National Food Institute, WHO Collaborating Center for Antimicrobial Resistance in Food borne Pathogens and Genomics and European Union Reference Laboratory for Antimicrobial Resistance, Technical University of Denmark, -2800 Kgs. Lyngby, DK Denmark

**Keywords:** *Salmonella* Dublin, Virulence, SPI-6, SPI-19, T6SS, Vi antigen, *PagN*, *Ggt*, Gifsy-2

## Abstract

**Background:**

*Salmonella enterica* serovar Dublin is a zoonotic infection that can be transmitted from cattle to humans through consumption of contaminated milk and milk products. Outbreaks of human infections by *S.* Dublin have been reported in several countries including high-income countries. A high proportion of *S.* Dublin cases in humans are associated with invasive disease and systemic illness. The genetic basis of virulence in *S.* Dublin is not well characterized.

**Methods:**

Whole genome sequencing was applied to a set of clinical invasive and non-invasive *S.* Dublin isolates from different countries in order to characterize the putative genetic determinants involved in the virulence and invasiveness of *S.* Dublin in humans.

**Results:**

We identified several virulence factors that form the bacterial invasome and may contribute to increasing bacterial virulence and pathogenicity including mainly Gifsy-2 prophage, two different type 6 secretion systems (T6SSs) harbored by *Salmonella* pathogenicity islands; SPI-6 and SPI-19 respectively and virulence genes; *ggt* and *PagN*. Although Vi antigen and the virulence plasmid have been reported previously to contribute to the virulence of *S.* Dublin we did not detect them in all invasive isolates indicating that they are not the main virulence determinants in *S.* Dublin.

**Conclusion:**

Several virulence factors within the genome of *S.* Dublin might contribute to the ability of *S.* Dublin to invade humans’ blood but there were no genomic markers that differentiate invasive from non-invasive isolates suggesting that host immune response play a crucial role in the clinical outcome of *S.* Dublin infection.

**Electronic supplementary material:**

The online version of this article (doi:10.1186/s12879-017-2628-x) contains supplementary material, which is available to authorized users.

## Background

Salmonellosis is one of the most common foodborne diseases worldwide. *Salmonella enterica* (*S. enterica*) causes a huge global burden of morbidity and mortality in humans. It is estimated that *Salmonella* serovars responsible for typhoid fever kill over 250,000 humans each year [1] while non-typhoidal *Salmonella* (NTS) serovars that are responsible for diarrhoeal illness cause 155,000 deaths annually [2]. Moreover, NTS might have adapted to cause invasive disease and systemic infections in humans; children, the elderly and immunocompromised and it is estimated that 680,000 people die every year as a result of infection by invasive NTS (iNTS) [3].

The most predominant iNTS serovars associated with systemic illness in humans are *S.* Typhimurium, *S.* Choleraesuis and *S.* Dublin [4, 5]. *S.* Dublin is specifically adapted to cattle [6] thus, people can be infected through contact with infected animals or consumption of contaminated food including raw milk and raw-milk cheese. Outbreaks of human *S.* Dublin infection have been reported in some European countries including Ireland and France [7, 8].

Several virulence factors have been identified within the human adapted serovars Typhi and Paratyphi [9, 10]. On the other hand, little is known about the molecular basis of virulence in iNTS more specifically *S.* Dublin. Our understanding of the genetic basis of invasiveness in *S.* Dublin is skewed by the fact that most studies have focused on the most common iNTS; *S.* Typhimurium in particular the highly invasive multidrug-resistant (MDR) *S.* Typhimurium of a distinct Multilocus Sequence Type (MLST), ST313 that has been associated with severe infections and deaths in humans in sub-Saharan Africa [11, 12].

The aim of this study is to characterise the invasome of *S.* Dublin and the virulence factors that might enable the bacteria to invade blood causing systemic illness. We therefore applied whole genome sequencing (WGS) to a set of *S.* Dublin isolates from different countries all over the world.

## Methods

### Bacterial strains and whole genome sequencing

A set of *S.* Dublin isolates from different countries (Table 1) submitted to Centre National de Référence des Salmonella, Institut Pasteur were selected for WGS. The set of isolates included 22 human invasive isolates; 19 isolates from blood and 2 isolates from urine in addition to one isolate from pus. For comparison, we included 6 clinical non-invasive isolates from stool and 7 veterinary isolates. We also included the original *S.* Dublin isolate isolated from the stool of a patient in Dublin, Ireland (WS247) in 1929 giving the name of Dublin serovar. Furthermore, the reference *S.* Dublin isolate; SARB13 isolated in France from cattle in 1982 [13] was also included in this study.

**Table 1:** *Salmonella* Dublin strains included in this study

Isolate ID	Country (isolation year)	Source	MLST	rMLST	Plasmid replicons profile
WS247	Ireland (1929)	Human stool	10	53	*inc*X1 and *inc*FII
SARB13	France (1982)	Cattle	73	ND	*inc*FII
92.9894	Burkina Faso (1992)	Human blood	10	53	*inc*X1 and *inc*FII
93.1086	France (1992)	Cattle	10	53	*inc*X1, *inc*FIA and *inc*FIB
93.3170	Cameron (1993)	Human blood	10	53	No plasmid replicons
93.5462	Mali (1993)	Human blood	10	53	*inc*X1, *inc*FII and *Inc*Q1
93.1557	Senegal (1993)	Human stool	10	53	No plasmid replicons
94.2023	France (1994)	Cattle	10	53	*inc*X1 and *inc*FII
94.8298	Togo (1994)	Human blood	10	53	*inc*X1 and *inc*FII
98.5329	France (1998)	Human blood	10	53	*inc*X1 and *inc*FII
99.5828	Benin (1999)	Human blood	2037	53	*inc*X1 and *inc*FII
99.6207	Benin (1999)	Human blood	10	53	*inc*X1 and *inc*FII
00.8531	France (2000)	Milk	10	53	*inc*X1 and *inc*FII
00.7892	France (2000)	Shellfish	10	53	*inc*X1 and *inc*FII
01.9588	France (2001)	Human blood	10	53	*inc*X1 and *inc*FII
01.9808	France (2001)	Human blood	10	53	*inc*X1 and *inc*FII
02.5213	Côte d’ivoire (2002)	Human blood	10	53	*inc*X1 and *inc*FII
02.1212	France (2002)	Milk	10	53	*inc*X1 and *inc*FII
02.1209	France (2002)	Pork meat	10	53	*inc*X1 and *inc*FII
02.9836	France (2002)	Human stool	10	53	*inc*X1 and *inc*FII
03.2892	France (2003)	Human urine	10	53	*inc*X1 and *inc*FII
04.4663	Cameron (2004)	Human blood	73	ND	*inc*X1 and *inc*FII
05.1078	France (2005)	Human blood	10	53	*inc*X1 and *inc*FII
05.6136	Mali (2005)	Human blood	10	53	*inc*X1 and *inc*FII
05.5914	Togo (2005)	Human stool	10	53	*inc*X1 and *inc*FII
05.2324	Peru (2005)	Human blood	10	53	*inc*X1, *inc*FII and *Inc*Q1
08.6645	Congo (2008)	Human blood	10	53	*inc*X1 and *inc*FII
09.2054	France (2009)	Human stool	10	53	*inc*X1 and *inc*FII
201,001,882	France (2010)	Human blood	10	53	*inc*X1 and *inc*FII
201,005,507	Nigeria (2010)	Human blood	10	53	No plasmid replicons
201,200,014	France (2012)	Human blood	10	53	*inc*X1 and *inc*FII
201,200,083	France (2012)	Human urine	10	53	*inc*X1 and *inc*FII
201,208,251	France (2012)	Human pus	73	ND	*inc*X1 and *inc*FII
201,200,586	Maurice island (2012)	Human stool	10	53	*inc*X1 and *inc*FII
201,208,243	Thailand (2012)	Human blood	10	53	*inc*X1 and *inc*FII

All *S.* Dublin isolates do not harbor ARGs except two isolates; 93.5462 harbours ARGs to aminoglycoside, phenicol and sulphonamide while the isolate 05.2324 harbours ARGs to aminoglycoside, beta-lactam, sulfonamide and trimethoprim

WGS was carried out by the Center for Genomic Epidemiology at the Technical University of Denmark where genomic DNA was prepared for Illumina pair-end (PE) sequencing using the Illumina (Illumina, Inc., San Diego, CA) NexteraXT® Guide 150,319,425,031,942 following the protocol revision C (http://support.illumina.com/downloads/nextera_xt_sample_preparation_guide_15031942.html). A sample of the pooled NexteraXT Libraries was loaded onto an Illumina MiSeq reagent cartridge using MiSeq Reagent Kit v2 and 500 cycles with a Standard Flow Cell. The libraries were sequenced using an Illumina platform and MiSeq Control Software 2.3.0.3. All isolates were pair-end sequenced using 100 bp PE libraries. Raw sequence data have been submitted to the European Nucleotide Archive (http://www.ebi.ac.uk/ena) under study accession no.: PRJEB17616 (http://www.ebi.ac.uk/ena/data/view/PRJEB17616).

### Bioinformatic analysis

The quality of the raw sequence data was evaluated using FastQC toolkit (http://www.bioinformatics.babraham.ac.uk/projects/fastqc/). Low quality reads were removed using ea.-utils package (https://expressionanalysis.github.io/ea-utils/). PE reads were assembled using Velvet [13] and the best possible assembly with the highest N50 value was annotated using RAST server [14]. The assembled sequences were analyzed to detect the sequence type (ST) of *S. enterica,* plasmid replicons and acquired antimicrobial resistance genes (ARGs) using MLST, PlasmidFinder and ResFinder respectively available from Center for Genomic Epidemiology (CGE) https://cge.cbs.dtu.dk//services/all.php. Ribosomal MLST (rMLST) was determined using Enterobase https://enterobase.warwick.ac.uk/

PE reads from each isolate were mapped against the reference genome of *S.* Dublin strain CT_02021853 (accession number: NC_011205.1) using Burrows Wheeler Aligner (BWA) [15]. Single nucleotide polymorphisms (SNPs) were identified using samtools mpileup [16]. The best-fit model for nucleotides substitution was determined by jModelTest [17] then a maximum likelihood (ML) phylogeny based on SNPs was constructed by MEGA6 software [18] using 1000 bootstrap replicates.

BLASTn [19] was used for the alignment of virulence genes and genomic regions. Blast Ring Image Generator (BRIG) was used to illustrate the presence/absence of the virulence determinants [20].

## Results

### Putative virulence regions and genes in S. Dublin

**Fig. 1:**
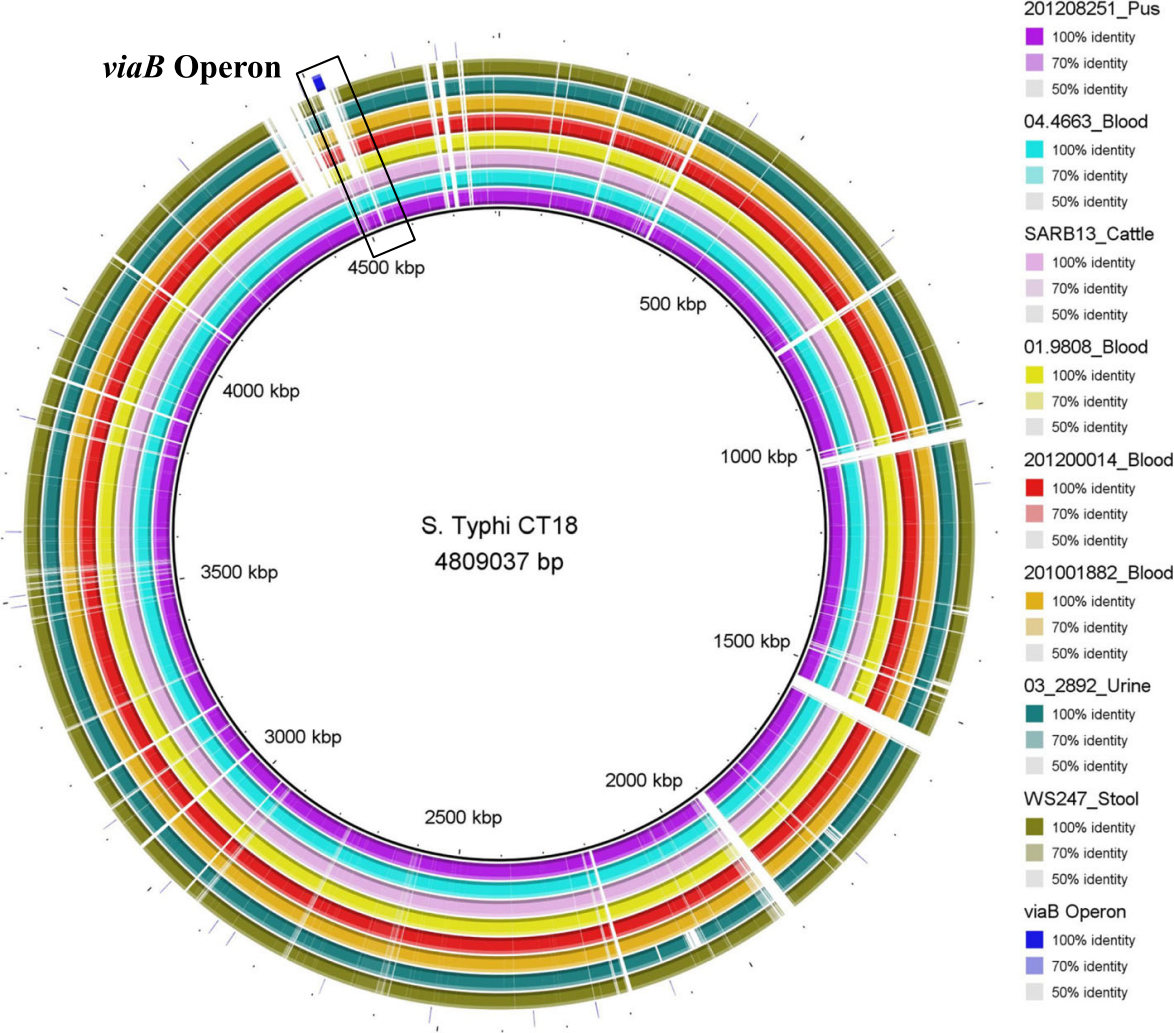
Complete genome alignment of *S*. Dublin isolates from France. The *viaB* operon is present in the reference *S.* Typhi str. CT18 (accession number: AL513382) but absent from all *S.* Dublin isolates sequenced in this study except three isolates including an isolate from human blood (04.4663), an isolate from human pus (201208251) and the reference SARB13 isolate from cattle

Vi-coding genes harboured by *Salmonella* Pathogenicity Island (SPI); SPI-7 were absent from all *S.* Dublin isolates except three isolates including the reference cattle isolate; SARB13 and two clinical isolates; 04.4663 from blood and 201,208,251 from pus (Fig. 1).

Interestingly, genes coding for flagellum biosynthesis were identical among invasive and non-invasive isolates.

**Fig. 2:**
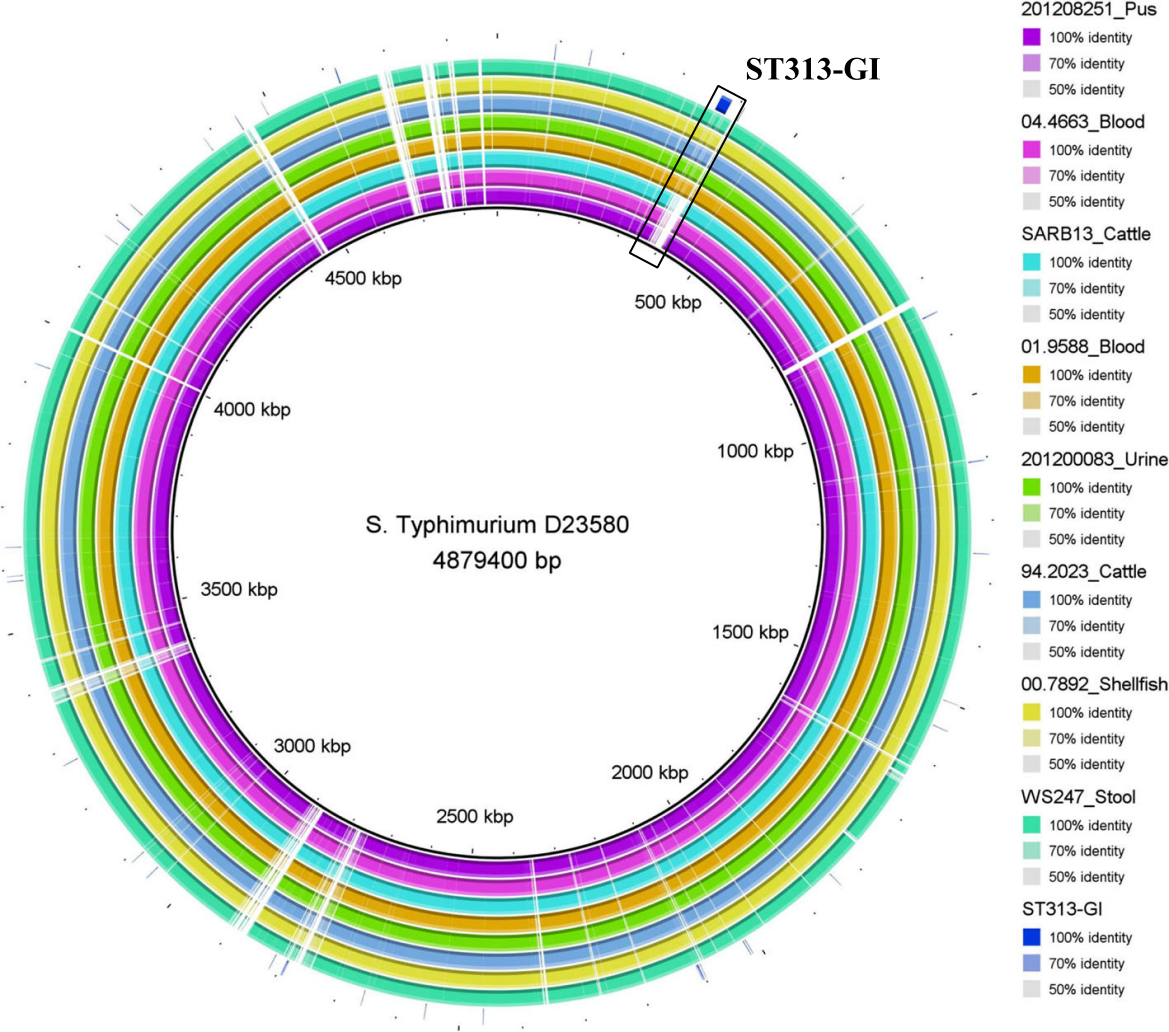
Complete genome alignment of *S*. Dublin isolates from France. The novel genomic island ST313-GI harbouring the gene *st313-td* is present in the invasive *S.* Typhimurium str. D23580 (accession number: FN4244051) however they are absent from the three Vi antigen positive *S.* Dublin isolates (04.4663, 201,208,251 and SARB13). The other *S.* Dublin isolates harbour the putative virulence gene *st313-td* despite that the genomic island ST313-GI is degraded

All *S.* Dublin isolates except these three isolates; SARB13, 04.4663 and 201,208,251 harbour the putative virulence gene *st313-td* on the degraded pathogenicity island ST313-GI (Fig. 2) which is entirely absent from the Vi positive three isolates (SARB13, 04.4663 and 201,208,251).

**Fig. 3:**
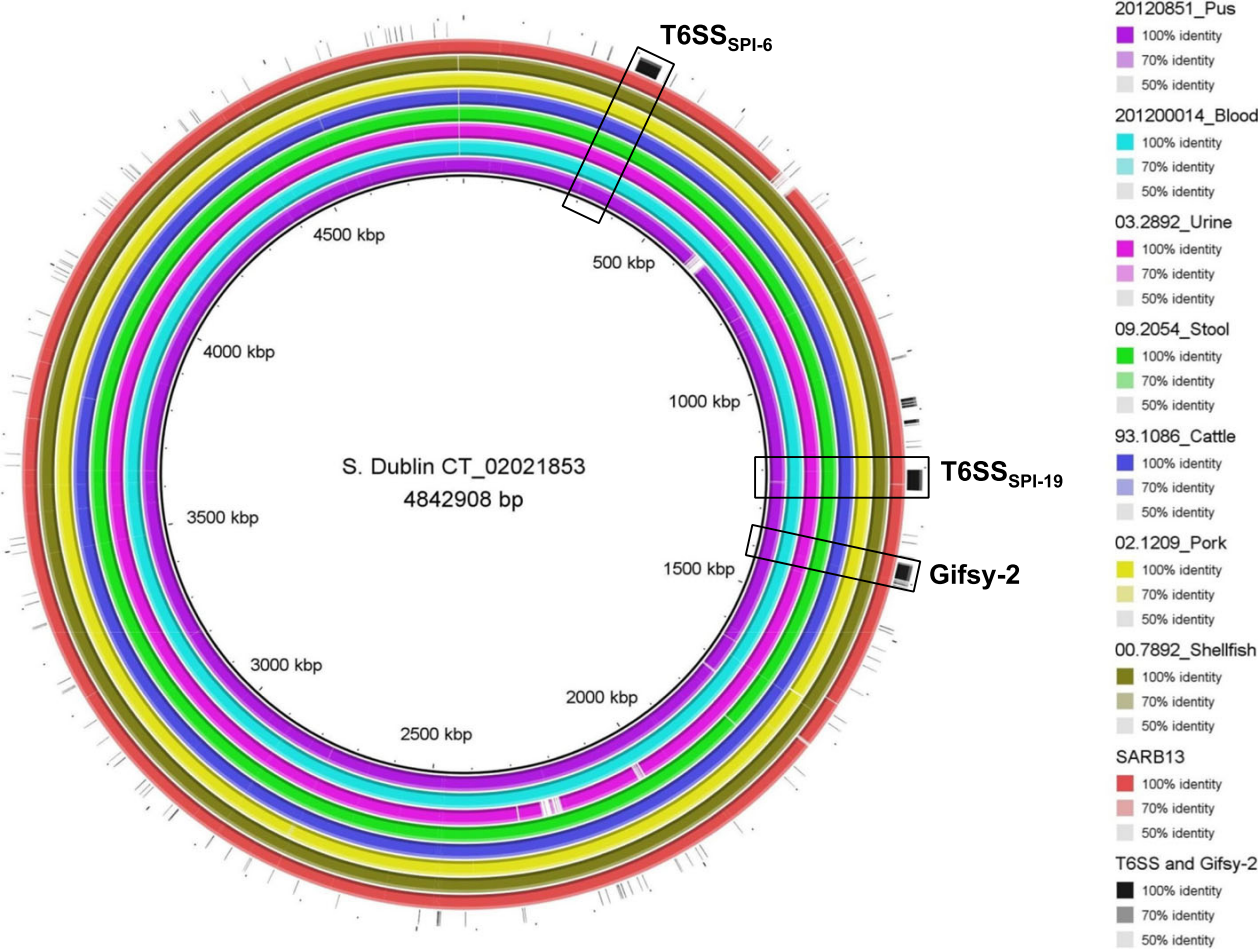
Complete genome alignment of *S*. Dublin isolates from France. *S.* Dublin str. CT_02021853 (accession number: NC_011205.1) is used as a reference. Gifsy-2 like prophage (accession number: NC_010393) and the two different T6SSs present in reference *S.* Dublin str. CT_02021853 including T6SS_SPI-6_ (Locus_tag: SeD_A0289 - SeD_A0326) and T6SS_SPI-19_ (Locus_tag: SeD_A1212 - SeD_A1243) are present in all clinical and veterinary isolates from France. The virulence plasmid pCT02021853_74 (accession number: NC_011204.1) was detected in all *S.* Dublin isolates expect three clinical isolates including 93.1557 from stool and 93.3170 and 201,005,507 from blood

On the other hand, all *S.* Dublin isolates sequenced in this study harbour pathogenicity islands SPI-6 and SPI-19 that encode type VI secretion system (T6SS); T6SS_SPI-6_ and T6SS_SPI-19_ respectively and they are all lysogenic for Gifsy-2 prophage (Fig. 3) that harbor the gene encoding Gifsy-2 prophage attachment and invasion protein.

An accessory genome that is identical to the virulence plasmid pCT02021853_74 (accession number: NC_011204.1) that harbor the virulence *spv* locus was detected in all *S.* Dublin isolates (plasmid replicons; *inc*X1 and *inc*FII) expect three clinical isolates; including 93.1557 from stool and 93.3170 and 201,005,507 from blood that harbour no plasmids altogether. On the other hand, the cattle isolate 93.1086 has a similar but smaller plasmid than the virulence plasmid pCT02021853_74 that lacks the virulence *spv* locus as a result of internal deletion.

Interestingly, the clinical invasive isolates; 93.5462 from Mali and 05.2324 from Peru harbour another plasmid; plasmid ST4/74 of *S.* Typhimurium (accession number: CP002490) and plasmid pSBLT of *S.* Typhimurium (accession number: LN794247) respectively in addition to the virulence plasmid pCT02021853_74 of *S.* Dublin.

All *S.* Dublin isolates including clinical and veterinary isolates harbor putative virulence factors including *ggt* and *PagN* genes that encode for γ-glutamyl transpeptidase (GGT) and an outer membrane protein respectively. The distribution of the putative virulence factors among all *S.* Dublin isolates are provided in Additional file 1: Table S1.

### Antimicrobial resistance genes present in S. Dublin isolates

No acquired ARGs were detected in *S.* Dublin isolates except in two clinical invasive isolates from blood including isolate 93.5462 isolated in 1993 from Mali that harbours ARGs to aminoglycoside (*strA, strB,* and *aadA1*), phenicol (*catA1*) and sulphonamide (*sul1* and *sul2*) on the plasmid ST4/74 of *S.* Typhimurium and isolate 05.2324 isolated from Peru in 2005 that has ARGs to aminoglycoside (*strA, strB,* and *aadA1* and *aac(3)-IIa*), beta-lactam (*blaTEM-1B*), sulfonamide (*sul1* and *sul2*) and trimethoprim (*dfrA1*) on the plasmid pSBLT of *S.* Typhimurium.

### Phylogenetic relationship among S. Dublin isolates

The phylogenetic SNP analysis showed that invasive and gastroenteritis isolates were intermixed as SNPs were randomly distributed around the chromosome of *S.* Dublin.

**Fig. 4:**
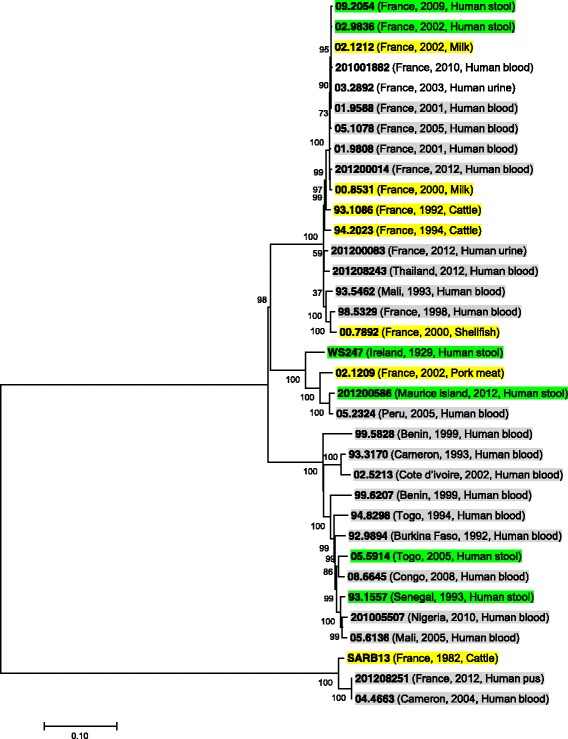
Maximum-likelihood phylogenetic tree of *S.* Dublin strains from different countries; invasive clinical isolates are highlighted in *grey*, gastroenteritis human isolates are highlighted in *green* and veterinary isolates are highlighted in *yellow*. The tree is based on SNPs determined from the whole genome sequence. Tree was inferred by using a general time-reversible (GTR) model. Bootstrap support values, given as a percentage of 1000 replicates, are shown above the branches

All isolates except three isolates; SARB13 from cattle, 201,208,251 from human pus and 04.4663 from human blood, were very closely related (Fig. 4), showed higher divergence from the other isolates and they have a distinct MLST (ST-73) and a distinct rMLST while other *S.* Dublin isolates have ST-10 except one isolate; 99.5828 that have ST-2037 but they all have the same rMLST; 53 (Table 1).

## Discussion

Human infection with iNTS represents a significant public health problem. A high proportion of *S.* Dublin cases in humans are characterized by bloodstream infection. Furthermore, the antibiotic resistance is increasing in *S.* Dublin as we detected two clinical isolates from blood that are resistant to multiple antibiotics.

The invasiveness of *S.* Dublin may be related to the expression of the Vi (virulence) antigen which is a capsular polysaccharide antigen commonly found in the human adapted *S.* Typhi and *S.* Paratyphi C. It has however also been detected in *S.* Dublin [21].

The Vi proteins are encoded within the *viaB* (Vi capsule biosynthesis) locus on SPI-7 [22, 23]. The *viaB* locus contains both Vi antigen biosynthetic genes (*tviB, tviC, tviD* and *tviE*) and export genes (*vexA, vexB, vexC, vexD* and *vexE*). The expression of Vi antigen is controlled by the *rcsB-rcsC* and *ompR-envZ* two-component regulator systems which lie outside the SPI-7 they however, interact with *tviA*; the first gene of the *viaB* gene cluster and regions upstream of the *tviA* promoter [24].

Experimental studies showed that Vi antigen is antiopsonic and antiphagocytic as it reduces the level of *S.* Typhi-induced tumor necrosis factor alpha (TNFα) by human macrophages and it also increases the resistance of *S.* Typhi to oxidative stress [25, 26].

In this study, we screened all *S.* Dublin isolates from France for the *viaB* operon and we found that Vi-coding genes were absent from all isolates except three isolates including the reference veterinary isolate; SARB13 and two clinical isolates; 04.4663 from blood and 201,208,251 from pus. We therefore conclude that it is unlikely that Vi antigen is the main virulence determinant for *S.* Dublin since it is absent from other invasive isolates from blood and urine.

Although Vi capsular polysaccharide antigen was not present in all invasive isolates it is possible that *S.* Dublin produces a unique O-antigen capsule that plays a role in increasing bacterial virulence and pathogenicity. Further in vitro and in vivo studies using O-antigen capsule deficient mutant are required to confirm this hypothesis.

Flagellum is considered as a virulence factor for motile bacteria such as *Salmonella* [27]. The surface domains of the flagellin protein are highly immunogenic and play an important role in triggering host innate and adaptive immune responses therefore, motile bacteria have evolved several mechanisms to overcome flagellin recognition by host receptors [28] however we detected no diversity among invasive an non-invasive *S.* Dublin strains in flagellar biosynthesis genes including *fliC* gene coding for flagellin, *fliD* coding for the flagellum capping protein and *fliA* coding for flagellum-specific sigma factor.

Although the three *S.* Dublin isolates; 04.4663, 201,208,251 and SARB13 are positive for the Vi antigen they lack the novel pathogenicity island ST313-GI that harbours the putative virulence gene *st313-td.* On the other hand, all other *S. Dublin* isolates harbour the *st313-td* gene on a degraded ST313-GI.

Although the exact role of *st313-td* in increasing bacterial virulence is not known experimental studies showed that *st313-td* is associated with the virulence and systemic infection in invasive *S.* Typhimurium of a distinct MLST, ST313, that has emerged recently in Africa [12]. It has been shown that virulence of *S*. Typhimurium in the mouse model was reduced in the absence of *st313-td* therefore, it is hypothesised that *st313-td* might help in bacterial evasion from host’s immune system through decreasing the binding of the bacteria to specific antibodies causing less uptake by macrophages or through decreasing the susceptibility to complement mediated lysis [11, 28].

We hypothesise that it is likely that *st313-td* plays a role in the virulence of *S.* Dublin hence, we detected it in all isolates (except the three isolates that are positive for Vi antigen; 04.4663, 201,208,251 and SARB13) however further experimental work is required to test its role in the virulence of *S.* Dublin.

There are other mobile genetic elements (MGEs) (excluding SPI-7 harbouring Vi antigen and genomic island ST313-GI encoding the putative virulence gene *st313-td*) that might also contribute to the virulence of *S.* Dublin in humans including SPI-6 and SPI-19 which harbour two different types of T6SS including T6SS_SPI-6_ and T6SS_SPI-19_ respectively.

It has been shown that T6SSs play a significant role in bacterial pathogenesis as they function as contractile nanomachines to puncture human cells and deliver virulence and lethal factors [29]. Experimental studies showed that T6SS_SPI-6_ plays a crucial role in the invasiveness and the systemic spread of *S.* Typhimurium [30] while T6SS_SPI-19_ has been reported to contribute to the survival of the poultry adapted serovar *S.* Gallinarum within infected macrophages [31].

All *S.* Dublin isolates harbour SPI-6 and SPI-19 that encode T6SS_SPI-6_ and T6SS_SPI-19_ respectively. Interestingly, SPI-6 and SPI-19 harbouring the two different T6SS have been also detected in clinical human isolates from Ireland including invasive and gastroenteritis isolates [32] suggesting that they contribute to the ability of *S.* Dublin to cause invasive disease in humans.

Another MGE that might contribute to the virulence and pathogenesis of *Salmonella* serovars including *S.* Dublin is the lambdoid prophage Gifsy-2 [33] as it encodes several virulence genes including *sodCI*, *sseI* and *gtgE* [34].

In this study, we found that all *S.* Dublin isolates are lysogenic for Gifsy-2 prophage and they all harbor the gene encoding Gifsy-2 prophage attachment and invasion protein suggesting that Gifsy-2 might be associated with the predisposition of *S.* Dublin to cause systemic illness in humans.

Plasmids are other MGEs that have been shown to play a significant role in the acquisition of virulence and antimicrobial resistance genes in several *Salmonella* serovars [35]. Experimental studies showed that *S.* Dublin plasmid encoding the virulence *spv* locus is involved in the dissemination and spread of bacteria to the blood [36, 37].

Interestingly, all *S.* Dublin isolates sequenced in this study except three clinical isolates including 93.3170 and 201,005,507 from blood and 93.1557 from stool harbour the virulence *S.* Dublin plasmid pCT02021853 that encodes the *spv* genes. Although the cattle isolate (93.1086) harbours a closely related plasmid to the virulence plasmid pCT02021853 the plasmid does not harbour the virulence *spv* locus. We therefore conclude that it is unlikely that the virulence plasmid is the main virulence determinant for *S.* Dublin and it is not critical for bacterial invasiveness since it is absent from two invasive clinical isolates from blood; 93.3170 and 05.1078.

Among the virulence factors that might contribute to the virulence of *S.* Dublin is γ-glutamyl transpeptidase (GGT) as it has been reported to contribute to the virulence of *Helicobacter pylori* [38] furthermore, it plays a significant role in inhibiting T-cell proliferation [39]. We found that the gene *ggt* is harboured by all *S.* Dublin isolates including human and animal isolates.

Another virulence gene, *PagN*, encodes for an outer membrane protein (PagN) that has been reported to contribute to the virulence of *S.* Typhimurium through mediating bacterial adhesion and invasion of mammalian cells [40, 41]. Experimental studies showed that PagN is a strong immuogen in mice and it can therefore be a potential vaccine candidate for salmonellosis [42]. Interestingly, we found that *PagN* is harboured by all *S.* Dublin isolates including invasive and non-invasive isolates suggesting its relation to bacterial virulence and invasiveness.

Although *S.* Dublin is adapted to cattle as a result of extensive genome decay and pseudogenes accumulation [6] *S.* Dublin can infect other animals [43, 44]. The phylogenetic SNP analysis showed the close relation among the veterinary isolates and human isolates as SNPs were randomly distributed around the chromosome of *S.* Dublin. All *S.* Dublin isolates were intermixed and there were no genomic differences among clinical invasive and non-invasive isolates.

## Conclusions

We identified several virulence factors in *S.* Dublin isolates that form the bacterial invasome however, we could not detect any genomic markers that differentiate invasive from non-invasive disease suggesting that host factors and immune response play a significant role in the disease outcome.

## Additional file

Additional file 1: Table S1. Distribution of putative virulence factors among *S*. Dublin isolates. (XLSX 13 kb)

## Abbreviations

ARG: Antimicrobial resistance gene
iNTS: Invasive nontyphoidal *Salmonella*
MGE: Mobile genetic element
NTS: Nontyphoidal *Salmonella*
*S.* Dublin: *Salmonella* Dublin
*S.* Typhimurium: *Salmonella* Typhimurium
SPI: *Salmonella* pathogenicity islands
T6SS: Type VI secretion system
